# Properties and Degradability of Poly(Butylene Adipate-Co-Terephthalate)/Calcium Carbonate Films Modified by Polyethylene Glycol

**DOI:** 10.3390/polym14030484

**Published:** 2022-01-26

**Authors:** Xiaoqian Diao, Caili Zhang, Yunxuan Weng

**Affiliations:** 1 Beijing Key Laboratory of Quality Evaluation Technology for Hygiene and Safety of Plastics, Beijing Technology and Business University, Beijing 100048, China; diaoxiaoqian@btbu.edu.cn; 2 College of Chemistry and Materials Engineering, Beijing Technology and Business University, Beijing 100048, China

**Keywords:** PBAT film, PBAT/CaCO_3_, coating agent, buried soil biodegradation, simulated seawater biodegradation

## Abstract

Poly(butylene adipate-co-terephthalate) (PBAT) is a biodegradable polymer synthesized from petrochemical resources. PBAT has an exceptionally high elongation at break values which makes it one of the most promising substitutes for LDPE packaging films. However, the applicability of PBAT films is still limited by low strength and high production costs. In this work, we used polyethylene glycol 600 (PEG-600) as a coating agent to modify the surface of calcium carbonate and improve compatibility with the polymer matrix. A series of PBAT/CaCO_3_ composite films having different CaCO_3_ particle size and content of coating agent was prepared using extrusion blow molding. The effect of particle size of CaCO_3_ filler and the content of a coating agent on the mechanical and rheological properties of composite films have been studied. The biodegradation properties have been tested by burying the samples in soil or keeping them in artificial seawater for 90 days. It was shown that the addition of PEG-600 improves compatibility between the matrix and CaCO_3_ filler as polar –OH groups of PEG have a high affinity toward the polar surface of CaCO_3_. Moreover, the hydrophilicity of PEG-600 increased the diffusivity of water molecules and facilitated PBAT degradation. This work provides experimental data and theoretical guidance that support the development of high-performance PBAT/calcium carbonate films for the single use packaging industry.

## 1. Introduction

Poly(butylene adipate-co-terephthalate) (PBAT) is a biodegradable plastic with excellent mechanical properties. Although synthesized from petrochemical resources, it has been considered as the most viable substitute for LDPE [1,2,3,4,5,6,7,8]. Although PBAT appeared on the market about a decade ago, the use of neat PBAT film products is limited by the high production costs and lower strength compared with LDPE as the most used packaging film material [9,10,11].

The mechanical properties of PBAT films are often reinforced by the incorporation of low-cost fillers such as starch, CaCO_3_, and hydrotalcite into the polymer matrix [12,13,14,15,16,17]. Calcium carbonate is one of the most applied inorganic fillers in thermoplastics [18,19,20,21,22]. The properties of polymer/CaCO_3_ composites depend on the amount of filler added to the polymer matrix, and the application type determines the maximum loading of CaCO_3_. For example, mulching films require good light transmittance and therefore tolerate only small amounts of CaCO_3_ [23]. On the other hand, packaging films could be filled with up to 30% CaCO_3_ to reduce the price of polymer composite while maintaining satisfactory mechanical properties [24]. The particle size of CaCO_3_, its dispersibility in the polymer matrix, and the affinity of a polymer toward filler determine the overall mechanical properties of a composite [25,26,27]. However, the main issue with this type of composite is the agglomeration that originates from the incompatibility between the filler and a polymer.

The addition of coating agents greatly enhances the stability of composite materials [28]. There are two types of coating agents classified according to the type of intermolecular interactions: (1) dispersants, which interact with particle surface through non-covalent Van der Waals forces, and (2) coupling agents, which are attached to the particle surface via covalent bonds. Fatty acids are the most widely used dispersants for CaCO_3_ and were applied in LDPE/CaCO_3_, HDPE/CaCO_3,_ and PP/CaCO_3_ composites [21,29,30]. Among coupling agents, silanes and titanates are often applied in the synthesis of composites [31].

We previously showed that the addition of a silane coupling agent effectively modified calcium carbonate and improved its compatibility with PBAT. Compared with neat PBAT/calcium carbonate films, the modified material had better mechanical properties and was more resistant to hydrolysis and photodegradation [32,33]. As mulching films should be stable during the long period of crop growth, the robustness and resistance to cracking and degradation of the silane-modified composite were beneficial for this purpose. However, polymer films for packaging and garbage bags have much shorter life cycle and require fast degradation once discarded in the environment. Therefore, we hoped that the addition of dispersant instead of coupling agent would improve the mechanical properties of PBAT/calcium carbonate composite films and accelerate biodegradation.

In this study, CaCO_3_ particles having different size (12, 6.5, and 5 μm) were modified with polyethylene glycol 600 (PEG-600). The effect of particle size and the amount of coating agent on mechanical and rheological properties of PBAT/CaCO_3_ composite films was studied in detail. Moreover, the biodegradation rate of these films was determined in soil and simulated seawater. It was anticipated that the hydrophilic PEG can increased the diffusivity of water molecules and facilitated PBAT degradation [34,35].

## 2. Experimental

### 2.1. Materials

PBAT (Ecoworld) was purchased from Jinhui Zhaolong Hi Tech Co., Ltd. (Shanxi, China). Three calcium carbonate powders with the particle size of 3000 mesh, 2300 mesh, and 1250 mesh were kindly supplied by Zhejiang Qintang Calcium Industry Co., Ltd. (Hangzhou, China). PEG-600 was purchased from Beijing Institute of Chemical Reagents Co. Ltd. (Beijing, China).

### 2.2. Preparation of PBAT/CaCO_3_ Composites

The composition of the mixtures prepared in this study is listed in Table 1. In a typical preparation procedure, CaCO_3_ was mixed with the coating agent in a high-speed mixer for 15 min to obtain surface-modified CaCO_3_. In the next step, dry PBAT was added, mixed thoroughly, and the mixture was transferred to the twin-screw extruder for melt blending, cooling, and granulation. Finally, the thin films were produced from composite pellets using the blowing film machine. More details about the preparation procedure can be found in previous work [32].

### 2.3. PBAT/CaCO_3_ Films Degradation in Simulated Seawater

To make an artificial seawater sample, the 2-cm thick bottom layer was prepared using the sand and mud from Ningbo Sea, and covered with the upper layer made of a 1:1 (*v*:*v*) mixture of artificial and natural seawater. Natural seawater was taken from Bohai Bay, PRC. The artificial seawater was prepared according to the previous reported work [36]. The temperature was held constant at 20 ± 2 °C during the degradation period of 90 days. The fresh aliquots were taken for analysis every 30 days.

### 2.4. The Degradation of PBAT/CaCO_3_ Films in Soil

**Figure 1:**
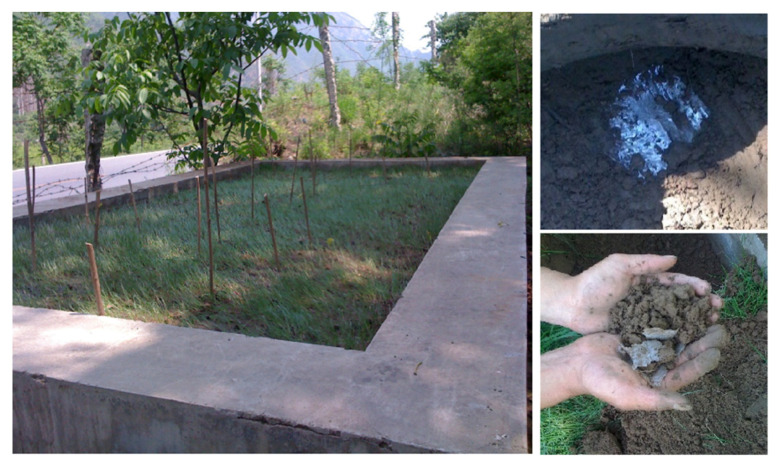
Soil biodegradation test site.

The soil degradation experiments were conducted by burying the samples at the lake coast in the western part of Beijing, PRC. More details about this procedure can be found in our previous publication [37]. The pictures of the degradation environment are shown in Figure 1. The soil degradation experiment was conducted from 1 July 2021 to 1 October 2021, 92 days in total. At the end of each month, the samples were taken out from the soil, cleaned, and dried at room temperature before characterization.

### 2.5. Characterization

A detailed procedure for structure characterization and performance evaluation is described in Supporting Information.

## 3. Results and Discussion

### 3.1. Effect of CaCO_3_ Particle Size and Coating Agent Content on the Properties of PBAT/CaCO_3_ Films

#### 3.1.1. Mechanical Properties

**Figure 2:**
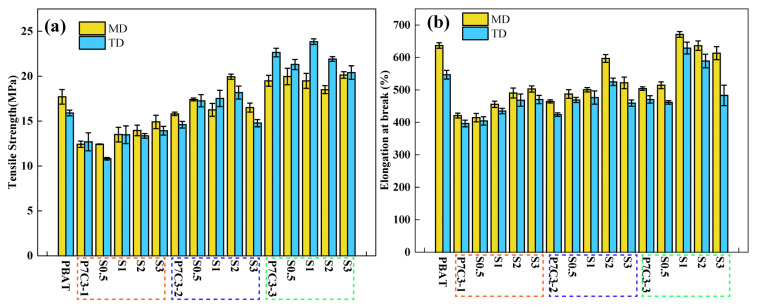
(**a**) Tensile strength and (**b**) elongation at break of PBAT/CaCO_3_ films.

Tensile strength, elongation at break, and tearing strength of PBAT composites filled with 30 wt.% of PEG-coated CaCO_3_ are listed in Table 2. It was shown that these mechanical properties depend on the particle size of a filler and the amount of coating agent. To better understand these trends, the variations in tensile strength and elongation at break have been showed in Figure 2. All properties of extruded films have been measured in the machine direction (MD) and the transverse direction (TD). The results revealed that the mechanical properties measured in both directions improve with the decrease of particle size from 12 μm to 5 μm. The superior performance of a sample P7C3-3 with the smallest particles is in accordance with the results of our previous work [32].

Comparing two samples having the same mass, the one with a smaller particle size has a larger surface area, and it could be expected that more coating agent is required to effectively modify its surface. However, it was interesting to observe that the amount of coating agent needed for the material to reach the optimum mechanical performance decreased with the particle size reduction. For example, the optimal content of the coating agent was 3% in P7C3-1, 2% in P7C3-2, and 1% in the P7C3-3 sample. This effect can be explained by strong electrostatic interactions between smaller CaCO_3_ particles that impede the dispersion and surface modification of individual particles. As a result of this process, an excess PEG coating agent remains trapped in the polymer matrix and represents another solid phase in the system. Being highly hydrophilic, it is not compatible with the hydrophobic PBAT matrix and the excess PEG decreases the mechanical properties of PBAT/CaCO_3_ composite films.

#### 3.1.2. Rheological Properties

**Figure 3:**
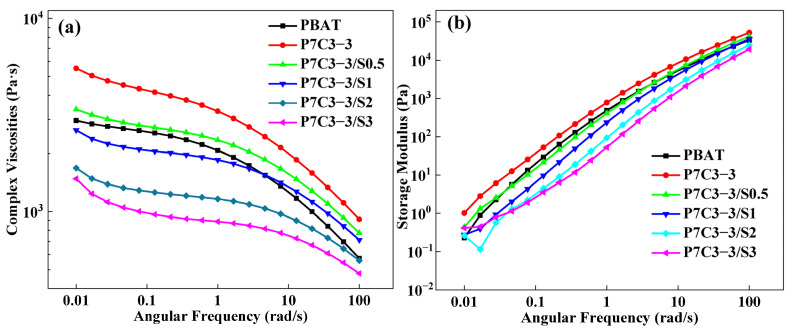
(**a**) Complex viscosity and (**b**) storage modulus of PBAT/CaCO_3_ films prepared using calcium carbonate with particle size 300 mesh and different amounts of PEG coating agent.

Rheological properties of molten composites strongly depend on the dispersibility of filler particles within the polymer matrix and the nature of intermolecular interactions. The effect of the coating agent on the structure and dispersion state of CaCO_3_ within the polymer matrix has been studied by measuring the complex viscosity and storage modulus of several composites modified by different amounts of PEG. These parameters have been compared with the values for neat PBAT (Figure 3). According to the results shown in Figure 3a, the viscosity of the modified P7C3-3 sample is higher than the value for neat PBAT. This happens due to formation of a “filler network” structure consisting of a significant amount of CaCO_3_ which agglomerates and aggregates [21]. This effect reduces the molecular mobility and free volume of polymer chains. Upon the addition of PEG, the complex viscosity drops in a concentration-dependent manner due to the lubricating effect of the coating agent.

The decreased mobility of PBAT chains also results in longer relaxation times and the increased storage modulus of PBAT/CaCO_3_ composites compared with neat polymer (Figure 3b). The addition of PEG coating agent improves the mobility of a polymer and decreases the storage modulus of P7C3-3 composites.

#### 3.1.3. Surface Morphology

The changes in the surface microstructure of PBAT films upon the addition of CaCO_3_ filler and PEG coating agent have been studied using SEM. The SEM images of modified and unmodified composite films captured at two magnifications are shown in Figure S1 (1000×) and Figure S2 (3000×). The surface of unmodified PBAT/CaCO_3_ films appeared relatively rough with abundant particle aggregates. Such a heterogeneous structure reduces the mechanical performances of material and might explain the results obtained in Section 3.1.1. On the other hand, composite films modified by the PEG coating agent exhibited relatively flat and smooth surface with good particle dispersion and without pronounced agglomeration. This confirms that the coating agent effectively increases the compatibility between PBAT and fillers.

### 3.2. Degradation Behavior of PBAT/CaCO_3_ Composites in Soil and Simulated Seawater Environment

The biodegradation of plastic films in soil or seawater is a complex process governed by the interplay of biological and physicochemical degradation mechanisms. According to the literature, three parameters govern the biodegradation rate of plastics: (1) the molecular structure and the thickness of polymer film; (2) the type and abundance of microbes in the environment, and (3) the physicochemical parameters of the environment including but not limited to temperature, acidity, humidity, and the presence of nutrients [38]. Previous studies have described that the biodegradation rate of samples buried in soil was mainly controlled by the soil microorganisms and moisture, where the microbial activity depends on temperature and moisture content in the soil [39]. The degradation processes that occur in seawater are somewhat different from those in the soil as seawater is rich in inorganic salts, deficient in organic nutrients, and has a weakly alkaline pH. In addition, the concentration of dissolved O_2_ is much lower in seawater compared with the soil environment which also influences the biodegradation rate of plastics [40]. In this study, we examined the biodegradation of PBAT/CaCO_3_ composites in soil and seawater.

#### 3.2.1. Visual Inspection of Structural Changes upon Biodegradation of PBAT/CaCO_3_ Composites in Soil and Simulated Seawater Environment

The initial thickness of the composite film was 0.055 ± 0.015 mm. After 30 days in the soil environment, we observed partial fragmentation and erosion holes, which was accompanied by the decrease of film thickness to 0.046 ± 0.018 mm. After another 30 days of environmental exposure, the composite films were essentially broken into fragments and the average film thickness was reduced to 0.029 ± 0.013 mm. The P7C3-2/S0.5, P7C3-2/S1, and P7C3-3 samples were almost completely degraded which complicated the sampling for the analysis. After 90 days in soil, all samples except P7C3-1, P7C3-3/S2, and P7C3-3/S3 were almost completely decomposed and the film thickness was reduced to 0.012 ± 0.008 mm.

**Figure 4:**
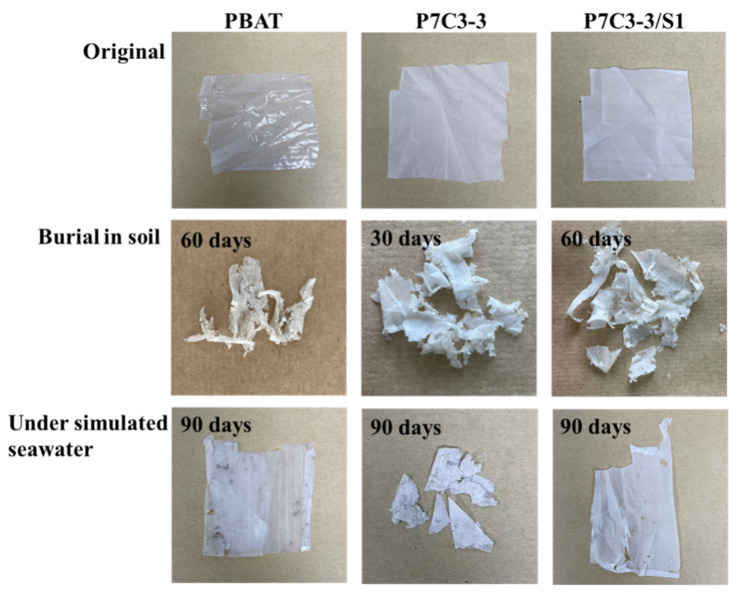
The optical images of neat PBAT, P7C3-3, and P7C3-3/S1 films before and after biodegradation in soil and simulated seawater.

The biodegradation processes in simulated seawater environment were much slower compared to soil. It took 60 days to observe the first changes in film thickness, and it was reduced from the initial value to 0.050 ± 0.012 mm. After 90 days in seawater, PBAT/CaCO_3_ composites changed the color to brown and some signs of fragmentation occurred. Moreover, the film thickness dropped to 0.044 ± 0.016 mm. The images of neat PBAT, P7C3-3, and P7C3-3/S1 films taken before and after biodegradation in soil and seawater are given in Figure 4.

#### 3.2.2. The Changes in Molecular Weight of Polymer upon Biodegradation Treatments

**Figure 5:**
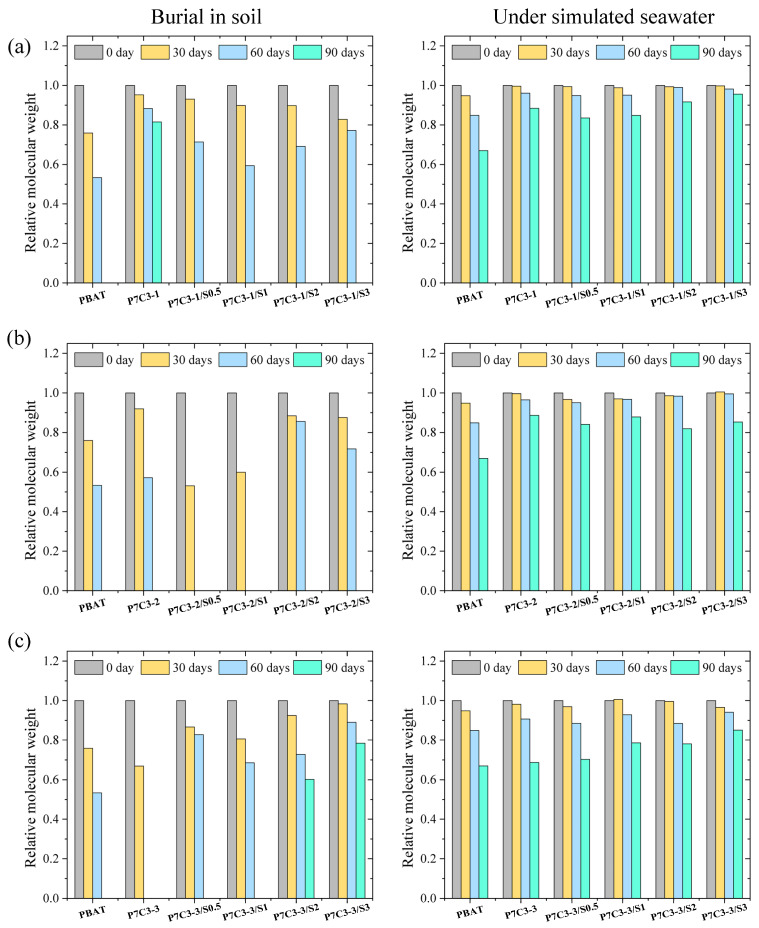
The relative changes in number molecular weight (*M*_n_) of neat PBAT, unmodified and coating agent modified PBAT/CaCO_3_ composites as a function of degradation time, expressed relative to day 0: (**a**) P7C3-1, (**b**) P7C3-2 and (**c**) P7C3-3.

The molecular weight (MW) of the polymer was measured to further characterize the decomposition of neat PBAT films and PBAT/CaCO_3_ composites after two biodegradation treatments mentioned before. The number average (*M*_n_) and weight-average MW (*M*_w_) of all films exposed to soil and seawater biodegradation for different periods are listed in Tables S1 and S2. The data from Tables S1 and S2 have been visualized in Figure 5 to observe how *M*_n_ changes with degradation time.

For all samples, *M*_n_ steadily decreased with soil degradation time. Comparing the degree of degradation for neat PBAT and PBAT composites with CaCO_3_ having various particle size, it decreased in the following order: P7C3-3 > P7C3-2 ≈ PBAT > P7C3-1. Two samples with the smallest particle size, namely P7C3-2 and P7C3-3 were almost completely decomposed after 90 and 60 days, respectively. This trend might be explained by the larger surface area of composite films filled with smaller CaCO_3_ particles, providing more opportunities for contact with water and oxygen molecules, thus facilitating the diffusion of water and hydrolysis/biodegradation of PBAT.

Next, we analyzed the effect of the coating agent on the extent of PBAT/CaCO_3_ degradation. It was found that the addition of an appropriate amount of PEG promotes decomposition, and this effect was most pronounced for P7C3-1 and P7C3-2 systems. The coating of CaCO_3_ with the hydrophilic PEG facilitates the diffusion of water molecules into the polymer matrix, which might explain the observed trends in degradation rate. This result is in accordance with the previous study that has demonstrated the increased water absorptivity of coating agent-modified PBAT/CaCO_3_ films [41]. Nevertheless, we observed that the degradation rate of PBAT decreases when the amount of coating agent exceeds 1%, regardless of CaCO_3_ particle size. For example, the addition of 2% or 3% of PEG coating agent added in P7C3-3 composite leads to incomplete degradation after 90 days in soil. This trend could be related to the previous discussion on the mechanical properties of PBAT/CaCO_3_ composites. When the amount of coating agent exceeds optimum, the dispersion of coated reinforcements being less successful, and the diffusion of water is less favored, this effect reduces the hydrolysis/biodegradation of the PBAT matrix. These results highlight the importance of the optimization of coating agent loading for mechanical and biodegradation film performances.

The molecular weight measurements also confirmed that the degradation rate in seawater is much slower than in soil. Although particle size and coating agent influence the diffusivity of water in the polymer matrix, it was found that the degradation rate of P7C3-3 and P7C3-3/S0.5 was similar to that of neat PBAT film. These results demonstrate the dominant role of biodegradation in the overall decomposition mechanism of PBAT/CaCO_3_ composites while hydrolysis has a minor influence. Some studies also revealed that the type and size of the microbial community are the key parameters for the biodegradation rate of plastics [42].

#### 3.2.3. FTIR Analysis of Changes in the Chemical Structure of PBAT upon Biodegradation

**Figure 6:**
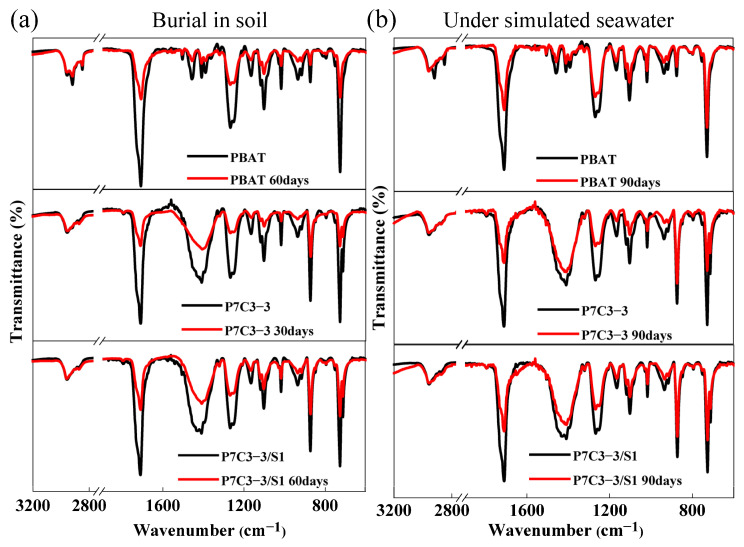
FTIR spectra of neat PBAT, P7C3-3, and P7C3-3/S1 films after 30, 60, or 90 days of biodegradation in (**a**) soil and (**b**) simulated seawater environment.

The changes in the structure of PBAT polyester caused by soil or seawater degradation have been studied by FTIR. The FTIR spectra of pure PBAT and different PBAT/CaCO_3_ composite films are shown in Figure 6. Two intense peaks observed at 1710 cm^−1^ and 1250 cm^−1^ originate from the stretching vibrations of carbonyl (C=O) and aliphatic C-O-C bonds of ester groups [43]. Soil degradation causes a significant decrease in the intensity of two ester peaks after 30 days of treatment, and the effect intensifies after the additional 30 days. Seawater biodegradation also reduces the intensity of FTIR ester peaks of PBAT but to a much lower extent. Moreover, the changes in FTIR spectra are observed in the fingerprint region. It can be concluded that the biodegradation of PBAT polyester proceeds mainly through the hydrolysis of ester bonds.

#### 3.2.4. The Changes in the Morphology of Polymers Caused by Biodegradation Revealed by SEM

**Figure 7:**
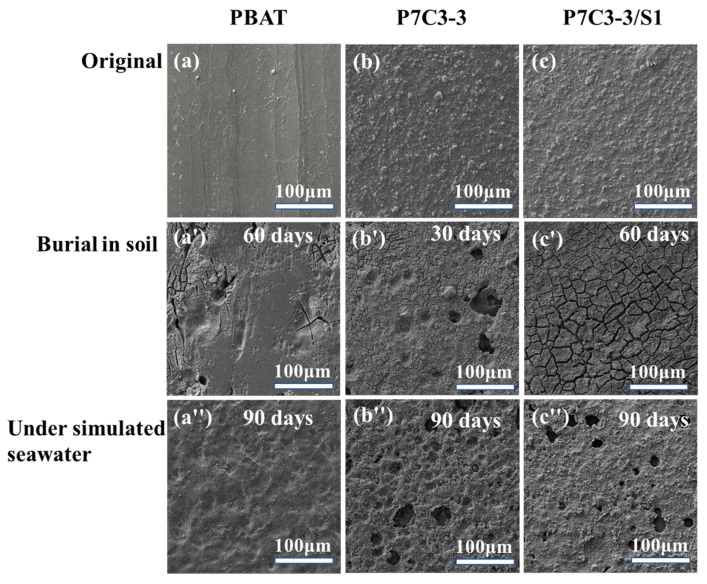
SEM images of (**a**) neat PBAT, (**b**) P7C3-3 and (**c**) P7C3-3/S1 films before and after biodegradation treatments. (**a’**–**c’**): burial in soil degradation; (**a”**–**c”**): under simulated seawater degradation.

The microstructure of neat PBAT, P7C3-3 composite, and composite with PEG-coated filler have been observed before and after each biodegradation test. According to SEM micrographs shown in Figure 7, the smooth surface of PBAT was preserved after 60 days of degradation in soil and only small pores and cracks occurred. Somewhat rougher surface of P7C3-3 films transformed into the structure with large pores and small cracks after 30 days in the soil environment. In case of surface-coated P7C3-3/S1 films, large cracks appeared at the surface after 60 days in soil.

The biodegradation in a simulated seawater environment resulted in significantly lesser changes in the microstructure of PBAT and composites. After 90 days of treatment, no visible signs of biodegradation have been observed in neat PBAT film. More pronounced structure defects appeared in P7C3-3 and P7C3-3/S1 films after 90 days in simulated seawater. The swelling of these polymers due to diffusion of water into the matrix resulted in the departure of CaCO_3_ particles and the occurrence of holes that could be observed from SEM images. In addition, no visible cracks were found for these samples. These results additionally support previous findings that CaCO_3_ as a filler and hydrophilic PEG as a coating agent promote the diffusion of water in a polymer matrix.

## 4. Conclusions

In this study, PEG was used as a dispersion agent for coating CaCO_3_ particles to improve mechanical properties and biodegradability of PBAT/CaCO_3_ composite films. Among three particle sizes of applied CaCO_3_ filler, the one with the smallest particles performed best in terms of mechanical and biodegradation properties. The addition of 1 wt.% of PEG coating agent further improved the performances of composite films. The results of biodegradation experiments in soil and simulated seawater revealed that the degradation is more pronounced in the soil environment, which highlights the importance of microbial activity in the overall mechanism of decomposition. The coating with PEG dispersant improved the compatibility between PBAT polymer and CaCO_3_ filler and facilitated the diffusivity of water molecules into the composite film resulting in faster degradation. After three months in soil, all PEG-modified samples were completely decomposed while the same period in seawater caused only minor structural changes. Using 3000 mesh CaCO_3_ particles and less than 1 wt.% of PEG coating agent gives a sample (P7C3-3/S1) with improved mechanical properties and similar degradation performance as neat PBAT. The developed composite is a promising substitute for LDPE as the low-cost, biodegradable single use packing material.

## Figures and Tables

**Table 1:** The composition of PBAT/CaCO_3_ materials prepared in this study.

Samples	PBAT (wt.%)	CaCO_3_ (wt.%)			PEG Coating Agent (wt.%) *
		1250 Mesh (12 μm)	2300 Mesh (6.5 μm)	3000 Mesh (5 μm)	
PBAT	100	-	-	-	-
P7C3-1	70	30	-	-	-
P7C3-1/S0.5	70	30	-	-	0.5
P7C3-1/S1	70	30	-	-	1
P7C3-1/S2	70	30	-	-	2
P7C3-1/S3	70	30	-	-	3
P7C3-2	70	-	30	-	-
P7C3-2/S0.5	70	-	30	-	0.5
P7C3-2/S1	70	-	30	-	1
P7C3-2/S2	70	-	30	-	2
P7C3-2/S3	70	-	30	-	3
P7C3-3	70	-	-	30	-
P7C3-3/S0.5	70	-	-	30	0.5
P7C3-3/S1	70	-	-	30	1
P7C3-3/S2	70	-	-	30	2
P7C3-3/S3	70	-	-	30	3

* The wt.% of coating agent relative to CaCO_3_.

**Table 2:** The influence of particle size of CaCO_3_ filler and the amount of coating agent on the mechanical properties of PBAT/CaCO_3_ composite films.

Sample Codes	Tensile Strength (MPa)		Elongation at Break (%)		Tearing Strength (KN/m)	
	MD	TD	MD	TD	MD	TD
PBAT	17.7 ± 0.81	15.9 ± 0.31	637 ± 8.5	547 ± 13.6	117.9 ± 5.21	110.8 ± 0.34
P7C3-1	10.4 ± 0.17	9.4 ± 0.38	481.1 ± 12.76	383.5 ± 13.31	75.1 ± 0.63	74.8 ± 3.14
P7C3-1/S0.5	12.42 ± 0.04	10.81 ± 0.14	414.57 ± 13.04	404.3 ± 13.12	87.8 ± 2.75	77.87 ± 1.61
P7C3-1/S1	13.5 ± 0.82	13.47 ± 0.99	455.77 ± 9.82	434.85 ± 8.28	93.41 ± 5.56	88.35 ± 3.91
P7C3-1/S2	13.96 ± 0.58	13.35 ± 0.26	490.61 ± 15.16	468.52 ± 18.90	93.36 ± 3.87	86.66 ± 3.24
P7C3-1/S3	14.91 ± 0.74	13.93 ± 0.49	502.84 ± 9.53	470.34 ± 12.65	95.7 ± 1.06	96.22 ± 2.58
P7C3-2	13.1 ± 1.71	13.4 ± 2.47	489.6 ± 11.32	466.0 ± 15.61	109.5 ± 1.64	115.9 ± 1.44
P7C3-2/S0.5	17.41 ± 0.16	17.26 ± 0.68	487.49 ± 13.38	469.30 ± 7.76	116.5 ± 1.28	115.06 ± 0.51
P7C3-2/S1	16.25 ± 0.72	17.52 ± 0.90	500.5 ± 6.70	476.58 ± 20.32	114.75 ± 5.37	118.36 ± 3.48
P7C3-2/S2	19.94 ± 0.29	18.18 ± 0.72	597.01 ± 11.90	524.93 ± 11.26	129.91 ± 1.83	125.4 ± 1.17
P7C3-2/S3	16.50 ± 0.50	14.77 ± 0.40	521.81 ± 17.92	459.42 ± 9.56	122.85 ± 2.95	117.65 ± 2.27
P7C3-3	15.6 ± 0.57	15.3 ± 0.30	547.6 ± 12.72	530.3 ± 6.70	116.9 ± 2.30	112.9 ± 1.42
P7C3-3/S0.5	19.97 ± 0.92	21.3 ± 0.56	514.54 ± 10.50	461.75 ± 5.26	137.73 ± 3.54	130.42 ± 2.94
P7C3-3/S1	19.48 ± 0.84	23.84 ± 0.32	670.97 ± 8.84	629.1 ± 18.49	141.14 ± 1.15	143.65 ± 1.67
P7C3-3/S2	18.5 ± 0.44	21.91 ± 0.26	636.38 ± 14.50	589.06 ± 20.68	127.46 ± 2.83	134.97 ± 0.27
P7C3-3/S3	20.13 ± 0.37	20.4 ± 0.76	613.34 ± 20.20	483.09 ± 31.67	117.54 ± 1.07	112.5 ± 2.69
P7C3-3/CA1-2 [32]	21.0 ± 0.51	20.3 ± 0.76	695.1 ± 10.28	625.8 ± 6.54	128.9 ± 2.37	127.3 ± 2.38

## Data Availability

The data presented in this study are available on request from the corresponding author.

## Supplementary Materials

The following supporting information can be downloaded at: https://www.mdpi.com/article/10.3390/polym14030484/s1, Characterizations; Figures S1 and S2: SEM images of composite films with different magnification; Table S1: Changes in molecular weight of PBAT/CaCO_3_ films under buried soil; Table S2: Changes in molecular weight of PBAT/CaCO_3_ films under simulated seawater degradation conditions.
