# Supplementary material for: Properties and Degradability of Poly(Butylene Adipate-Co-Terephthalate)/Calcium Carbonate Films Modified by Polyethylene Glycol
Source: Polymers (Basel). 2022 Jan 26;14(3):484. doi: 10.3390/polym14030484 (PMC8838338; doi:10.3390/polym14030484)
Supplement: Supplementary file 1 [file polymers-14-00484-s001.zip › polymers-1569932-supplementary.pdf]

Supporting Information

Properties and degradability of poly(butylene adipate-co-terephthalate)/calcium carbonate films modified by polyethylene glycol

Xiaoqian Diao[1,2], Caili Zhang[*1,2], Yunxuan Weng[*1,2]

[1] Beijing Key Laboratory of Quality Evaluation Technology for Hygiene and Safety of Plastics, Beijing Technology and Business University, Beijing 100048, China.

[2] College of Chemistry and Materials Engineering, Beijing Technology and Business University, Beijing 100048, China.

***** Correspondence: zhangcaili@btbu.edu.cn; wyxuan@th.btbu.edu.cn.

Characterizations:

The chemical structure of coating agent and degradation films was analyzed by Fourier transform infrared spectroscopy (FTIR) within performed on a Nicolet iN10 spectrometer (ThermoFisher Scientific) with 4 cm^{-1} resolution and 32 scanning frequency in the range of 400–4000 cm^{-1}. The chemical structure of coating agent was also confirmed by1H NMR spectra using $CDCl_3$ as the solvent (Bruker Avance 400 M).

Before testing the mechanical properties, the prepared samples were placed in the laboratory environment conditioning for 24 h. The tensile characteristics of the specimens were evaluated using a microcomputer-regulated electronic universal testing machine (Mester Industrial Systems Limited Corporation, CMT6104, China), according to the standard of GB/T 1040–2006. During the test, the tensile speed was 200 mm/min whereas the distance between the clamps was set as 50 mm. Individual groups of samples were tested five times, and the average value was taken according to the effective value. Using the same machine, the tear resistance properties of the samples were tested according to the standard of GB/T 106578.1–2008. During the test, the distance between the clamps was set as 50 mm and the tearing speed was kept at 100 mm/min. Individual groups of samples were tested five times, and the average value was taken according to the effective value.

The rheological characteristics of the composites were determined using a plate rheometer (Anton Paar, MCR502, Austria). The storage modulus, and complex viscosity of the composites were measured. The test temperature was 170°C, and 19

points were selected in the range of 0.01–100 rad/s.

The surface morphology of the films was observed by scanning electron microscope (FEI company, USA, Quanta 250 FEG) at 1000 times magnification. The accelerating voltage used in the scanning process was 10 kV.

Gel permeation chromatography was used to assess the molecular weight of the degradation films (GPC, 20A, Shimadzu, Japan). To make a 0.2 mg/ mL solution, 2 mg of fully dried material was dissolved in 10 mL dichloromethane. Then, the obtained solution was filtered with a polytetrafluoroethylene (PTFE) filter to remove impurities. As a mobile phase, dichloromethane was used at a flow rate of 2 mL/min. The molecular weight calibration curve was created using a variety of monodisperse polystyrene standards with molecular weights ranging from 3000 to 100,000 kDa. Then the molecular weight of the sample was obtained.

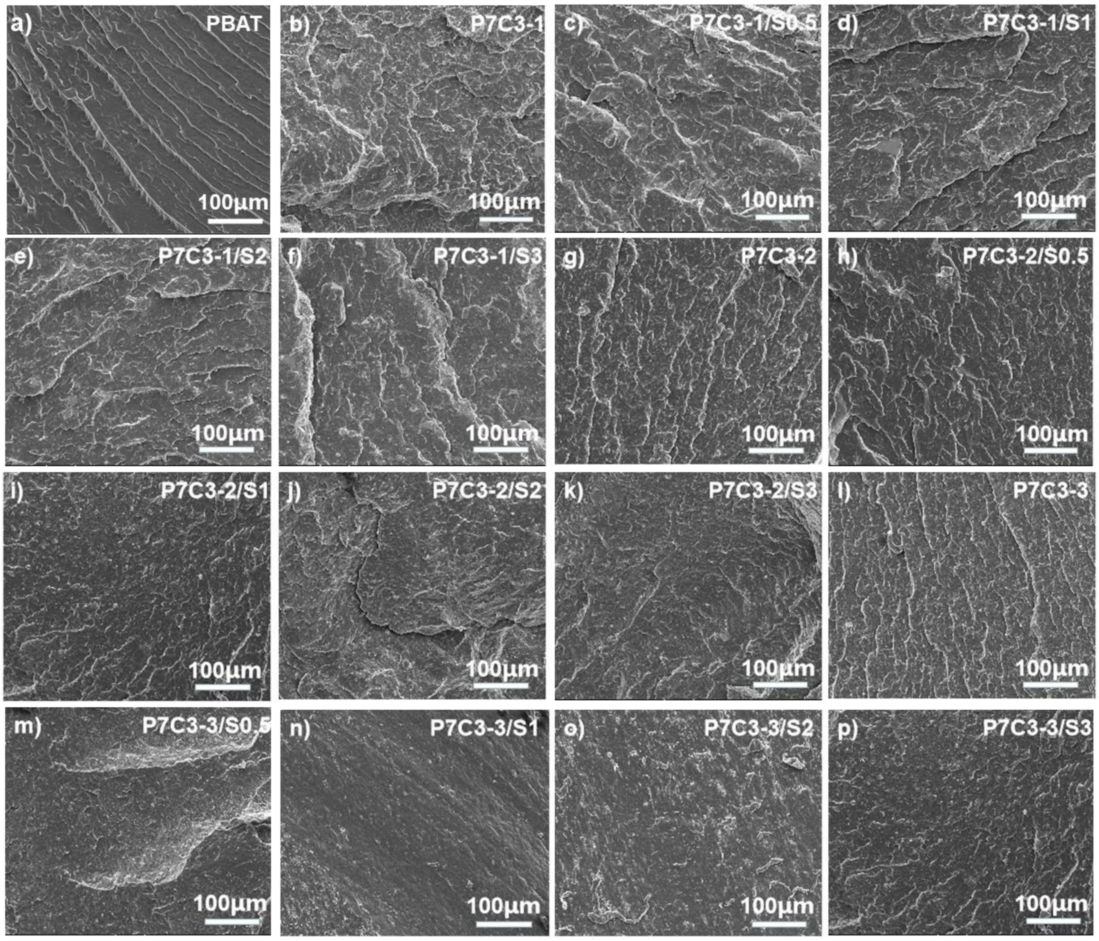

Figure S1 SEM images of films (×1000): (a) neat PBAT, (b) P7C3-1, (c) P7C3-1/S0.5, (d) P7C3-1/S1, (e) P7C3-1/S2, (f) P7C3-1/S3; (g) P7C3-2, (h) P7C3-2/S0.5, (i) P7C3-2/S1, (j) P7C3-2/S2, (k) P7C3-2/S3; (l) P7C3-3, (m) P7C3-3/S0.5, (n) P7C3-3/S1, (o) P7C3-3/S2, (p) P7C3-3/S3.

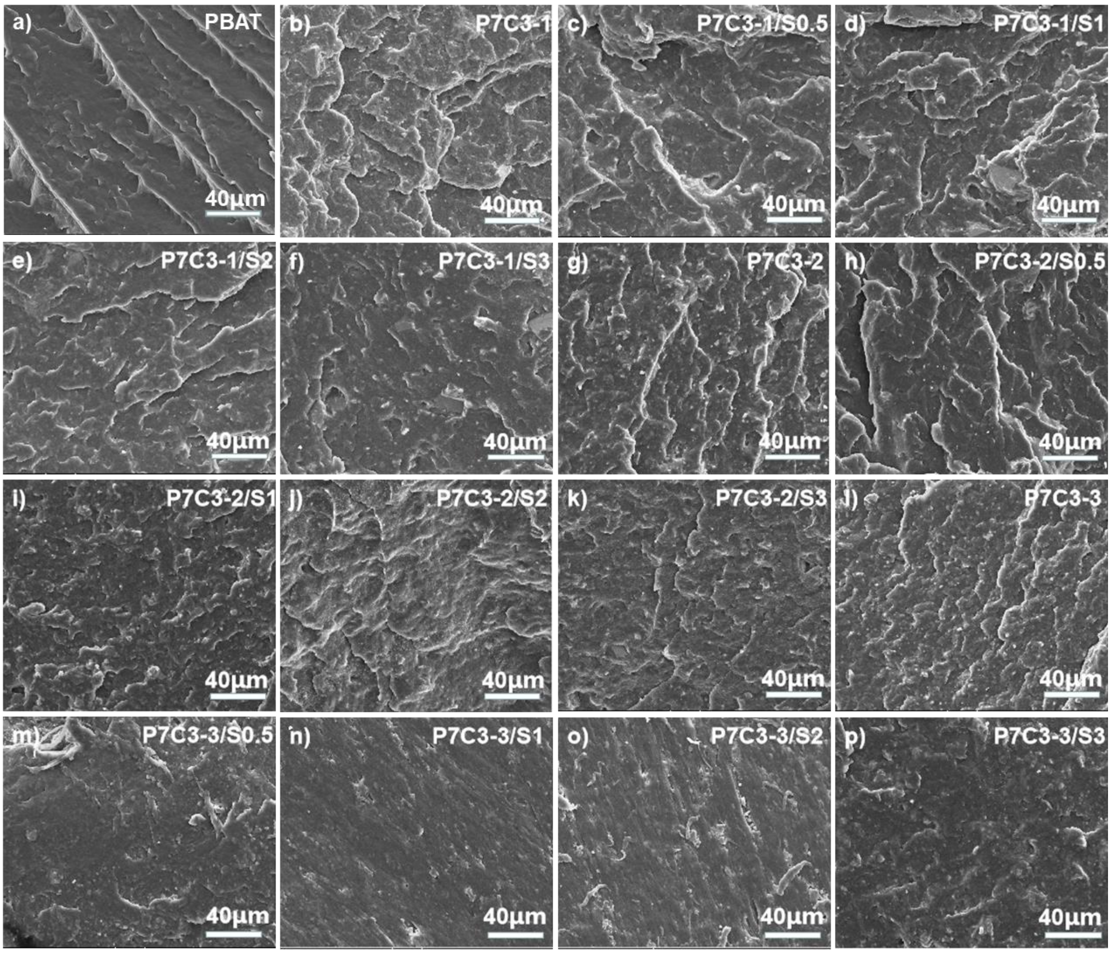

Figure S2 SEM images of films (×3000): (a) neat PBAT, (b) P7C3-1, (c) P7C3-1/S0.5, (d) P7C3-1/S1, (e) P7C3-1/S2, (f) P7C3-1/S3; (g) P7C3-2, (h) P7C3-2/S0.5, (i) P7C3-2/S1, (j) P7C3-2/S2, (k) P7C3-2/S3; (l) P7C3-3, (m) P7C3-3/S0.5, (n) P7C3-3/S1, (o) P7C3-3/S2, (p) P7C3-3/S3.

Table S1 Changes in molecular weight of PBAT/$CaCO_3$ films under buried soil

Sample codes	0 days molecular weight		30 days molecular weight		60 days molecular weight		90 days molecular weight	
	M_n	M_w	M_n	M_w	M_n	M_w	M_n	M_w
PBAT	63514	98339	48209	93002	33888	94077	--	--
P7C3-1	57146	98275	54425	95770	50444	99184	46580	85049
P7C3-1/S0.5	54254	99790	50504	94084	38737	86734	--	--
P7C3-1/S1	52607	97634	47275	98797	31265	81616	--	--
P7C3-1/S2	52080	96503	46754	82819	36000	82945	--	--
P7C3-1/S3	50178	88744	41569	97109	38746	84788	--	--
P7C3-2	57052	106400	52481	105147	32648	88034	--	--
P7C3-2/S0.5	52998	102524	28155	80735	--	--	--	--
P7C3-2/S1	52942	98754	31740	84944	--	--	--	--
P7C3-2/S2	53622	98065	47430	93315	45902	90017	--	--
P7C3-2/S3	50002	91734	43763	88303	35899	82005	--	--
P7C3-3	58816	102590	39358	100613	--	--	--	--
P7C3-3/S0.5	58014	102438	50271	96016	48009	92742	--	--
P7C3-3/S1	54416	96075	43860	90667	37291	85384	--	--
P7C3-3/S2	54295	95732	50242	91201	39519	87239	32690	82163
P7C3-3/S3	50096	92205	49272	89227	44612	86435	39330	83485

Table S2 Changes in molecular weight (g/mol) of PBAT/$CaCO_3$ films under simulated seawater degradation conditions

Sample codes	Original films		After 30 days		After 60 days		After 90 days	
	M_n	M_w	M_n	M_w	M_n	M_w	M_n	M_w
PBAT	63514	98339	60215	96722	53909	111460	42531	94057
P7C3-1	57146	98275	56906	98852	54902	98242	50532	93333
P7C3-1/S0.5	54254	99790	53905	10027	51464	92942	45323	92519
P7C3-1/S1	52607	97634	52003	103590	50014	100842	44626	98123
P7C3-1/S2	52080	96503	51711	94901	51532	99921	47761	95893
P7C3-1/S3	50178	88744	50068	91863	49255	98750	47947	96889
P7C3-2	57052	106400	56827	107090	55040	107914	50560	94473
P7C3-2/S0.5	52998	102524	51264	99873	50372	107362	44561	101059
P7C3-2/S1	52942	98754	51349	96157	51248	102649	46497	99744
P7C3-2/S2	53622	98065	52890	94464	52772	99637	43945	93705
P7C3-2/S3	50002	91734	50238	92378	49755	96238	42650	90735
P7C3-3	58816	102590	57716	103953	53344	94952	40399	94148
P7C3-3/S0.5	58014	102438	56251	105369	51364	103388	40786	101168
P7C3-3/S1	54416	96075	54765	97778	50539	96346	42795	99594
P7C3-3/S2	54295	95732	54082	94793	48052	95268	42420	97557
P7C3-3/S3	50096	92205	48364	89691	47137	93759	42614	90300
